# Crystal structures of two 6-(2-hy­droxy­benzo­yl)-5*H*-thia­zolo[3,2-*a*]pyrimidin-5-ones

**DOI:** 10.1107/S2056989015011044

**Published:** 2015-06-13

**Authors:** Ligia R. Gomes, John Nicolson Low, Fernando Cagide, Fernanda Borges

**Affiliations:** a FP–ENAS–Faculdade de Ciências de Saúde, Escola Superior de Saúde da UFP, Universidade Fernando Pessoa, Rua Carlos da Maia, 296, P-4200-150 Porto, Portugal; b REQUIMTE/Departamento de Química e Bioquímica, Faculdade de Ciências, Universidade do Porto, 4169-007 Porto, Portugal; c Department of Chemistry, University of Aberdeen, Meston Walk, Old Aberdeen AB24 3UE, Scotland; d CIQUP/Departamento de Química e Bioquímica, Faculdade de Ciências, Universidade do Porto, 4169-007 Porto, Portugal

**Keywords:** crystal structure, thia­zole, conformation, supra­molecular structure, hydrogen bonding, π–π stacking inter­actions

## Abstract

The title compounds, 6-(2-hy­droxy­benz­yl)-5*H*-thia­zolo[3,2-*a*]pyrimidin-5-one, C_13_H_8_N_2_O_3_S, (**1**), and 6-(2-hy­droxy­benz­yl)-3-methyl-5*H*-thia­zolo[3,2-*a*]pyrimidin-5-one, C_14_H_10_N_2_O_3_S, (**2**), were synthesized when a chromone-3-carb­oxy­lic acid, activated with (benzotriazol-1-yl­oxy)tripyrrolidinyl­phospho­nium hexa­fluorido­phosphate (PyBOP), was reacted with a primary heteromamine. Instead of the expected amidation, the unusual title thia­zolo­pyrimidine-5-one derivatives were obtained serendipitously and a mechanism of formation is proposed. Both compounds present an intra­molecular O—H⋯O hydrogen bond, which generates an *S*(6) ring. The dihedral angles between the heterocyclic moiety and the 2-hydroxybenzoyl ring are 55.22 (5) and 46.83 (6)° for (**1**) and (**2**), respectively. In the crystals, the mol­ecules are linked by weak C—H⋯O hydrogen bonds and π–π stacking inter­actions.

## Abstract 2

The title fused heterocycles arose from an unexpected intra­molecular cyclization reaction. Each mol­ecule features an intra­molecular O—H⋯O hydrogen bond. In the crystal, chains mediated by C—H⋯O inter­actions arise.

## Chemical context   

Although heterocycles, namely those bearing thia­zole or pyrimidine motifs, are reported to show a broad spectrum of pharmacological properties such as anti­microbial, anti­cancer and anti-inflammatory activities (Jiang *et al.*, 2013 ▸; Mishra *et al.*, 2015 ▸; Perrone *et al.*, 2012 ▸), only a few compounds enclosing the thia­zolo[3,2*a*]pyrimidine framework have been explored and screened towards the above-mentioned pharmacological activities. Even though some derivatives tested up to now have shown inter­esting anti-inflammatory (Bekhit *et al.*, 2003 ▸), anti­viral (Abd El-Galil *et al.*, 2010 ▸) and anti­bacterial activities (Mulwad *et al.*, 2010 ▸) and as calcium agonists (Balkan *et al.*, 1992 ▸), the data acquired so far are insufficient to indicate the importance of the thia­zolo[3,2*a*]pyrimidine motif as a positive contributor to the biological profile mentioned above. The same reflection is valid in relation to the data acquired for some thia­zolo[3,2*a*]pyrimidine-5-one derivatives as 5-HT2a receptor antagonists, a putative therapeutic target for the treatment of depression, although they have structural similarity to ritanserin, a serotonin antagonist (Awadallah, 2008 ▸). In this last case, the pharmacological activity appears to be enhanced by the nature of the planar aromatic or heterocyclic ring systems, the type of spacer as well as the presence of a basic nitro­gen atom.

A search made in the latest version (5.36.0; 2015) of the Cambridge Structural Database (Groom & Allen, 2014 ▸) for thia­zolo[3,2*a*]pyrimidine-5-one-based structures revealed the existence of 11 compounds containing the 5*H*-thia­zolo[3,2*a*]-pyrimidine-5-one fragment in which the hetero ring was not fused with other cyclic rings. In order to clarify the significance of the thia­zolo[3,2*a*]pyrimidine scaffold in medicinal chemistry, new 5*H*-thia­zolo[3,2-*a*]pyrimidin-5-one derivatives were synthesized. In this work we report the structures and synthesis, by a one-pot reaction, of two deriva­tives 6-(2-hy­droxy­benz­yl)-5*H*-thia­zolo[3,2-*a*]pyrimidin-5-one (**1**) and 6-(2-hy­droxy­benz­yl)-5*H*-thia­zolo[3,2-*a*]pyrimidin-3-methyl-5-one (**2**), which will be screened for anti­microbial activity.
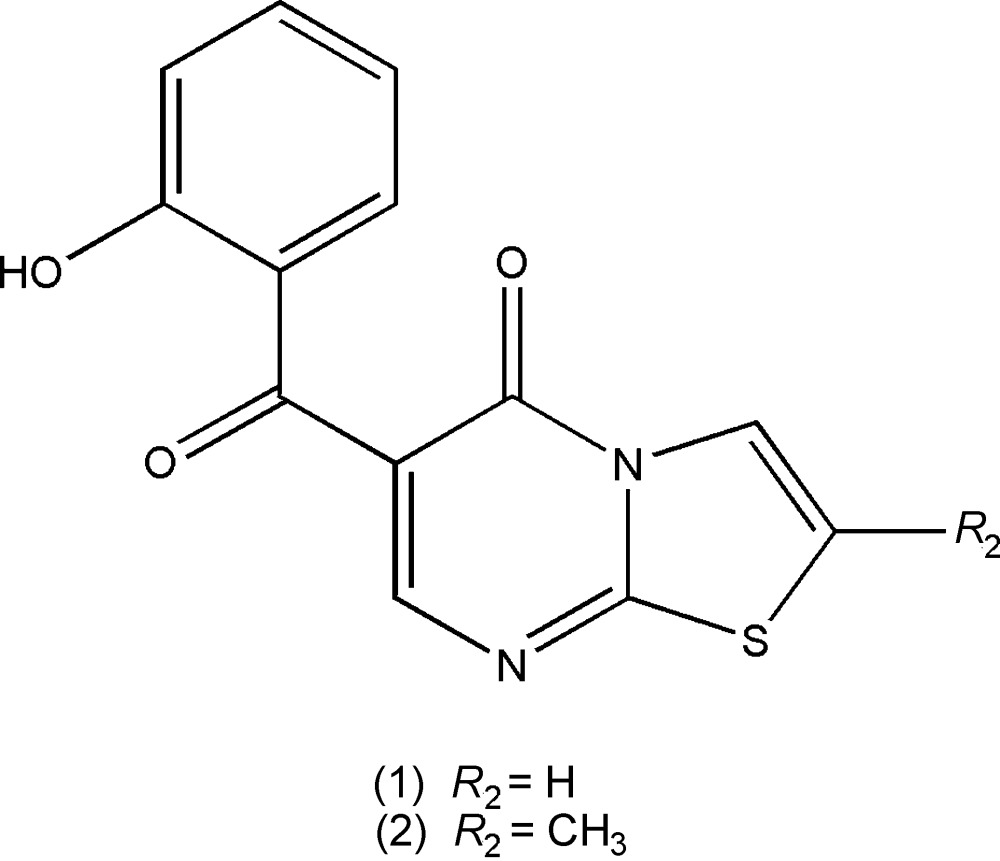


## Structural commentary   

**Figure 1:**
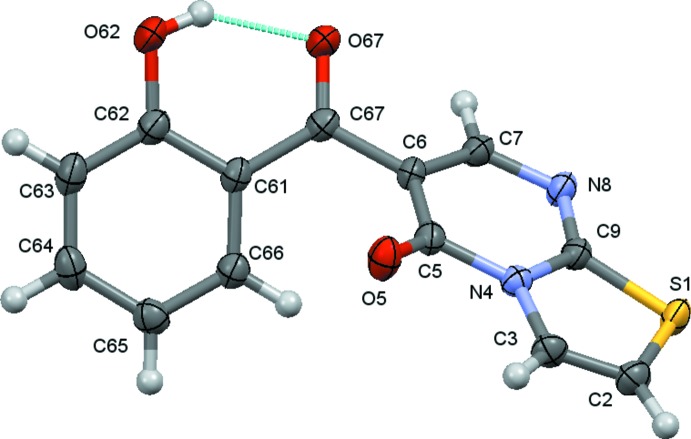
A view of the asymmetric unit of (**1**) with displacement ellipsoids drawn at the 70% probability level.

The mol­ecules of (**1**) and (**2**) are shown in Figs. 1 ▸ and 2 ▸. The structural characterization reveals that the mol­ecules have two cyclic units, *viz*. the hy­droxy­benzyl and the heterocyclic 5*H*-thia­zolo[3,2-*a*]pyrimidin-5-one ring separated by a carbonyl spacer, as expected. In both compounds, the carbonyl O atoms are *trans* oriented with respect to each other, contributing to the establishment of an intra­molecular O—H⋯O hydrogen bond between the *o-*hydroxyl group of the benzene ring and the carbonyl group of the spacer (Tables 1 ▸ and 2 ▸), which generates an *S*(6) ring. Taken together, the benzene ring and hydrogen-bonded pseudo ring are roughly planar, the carbonyl oxygen atom deviates by 0.391 (3) and 0.055 (4) Å in (**1**) and (**2**), respectively from the least-square plane formed by the benzene ring atoms. The heterocyclic rings of both compounds are also almost planar, as expected; the maximum deviation from the best plane formed by the ten atoms of the thia­zolo­pyrimidine moiety is 0.103 (1) Å for the carbonyl oxygen atom, O5, in (**1**) and 0.129 (1) Å for the same atom in (**2**). Thus, both mol­ecules are twisted around the C6—C67 bond that links the ring systems, which are inclined to one another by 55.22 (5) and 46.83 (6)° for (**1**) and (**2**), respectively.

## Supra­molecular features   

As noted above, the hydroxyl group is involved in intra­molecular hydrogen bonding, which leaves it unavailable for participation in inter­molecular hydrogen bonding. Thus, the mol­ecules are linked *via* weak C—H⋯O inter­actions: in both compounds the oxygen acceptor atom is the oxo atom O5, being in (**1**) the hydrogen-bond donor atom is C2 (of the heterocyclic group) and in (**2**) the hydrogen-bond donor atom is C64 (located in the exocyclic benzene ring).

**Figure 3:**
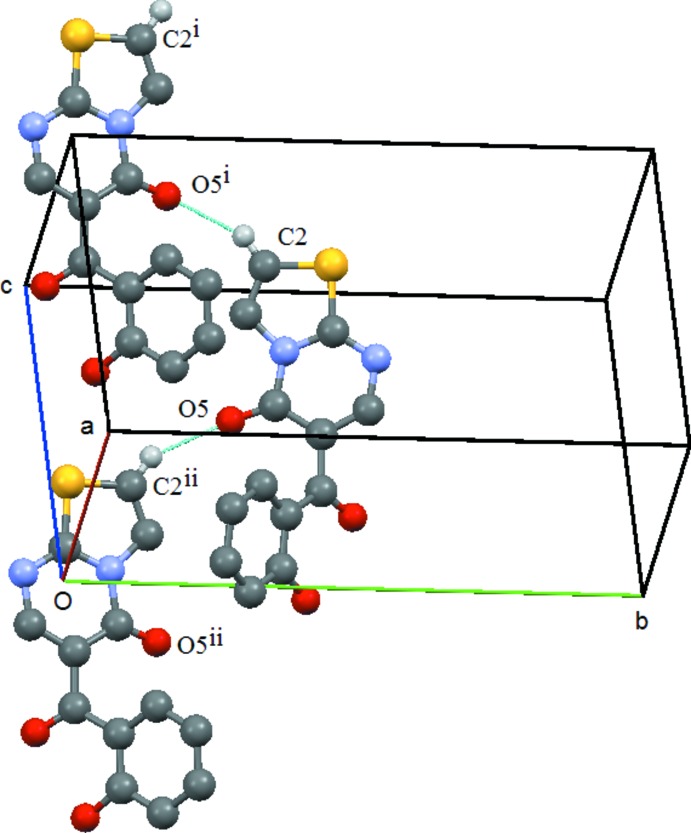
Compound (**1**): Mol­ecular *C*6 chain which runs parallel to [101]. Symmetry codes: (i) *x* + 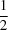, −*y* + 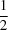, *z* + 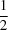; (ii) *x* − 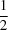, −*y* + 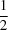, *z* − 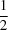. Hydrogen atoms not involved in the hydrogen bonding are omitted.

**Figure 4:**
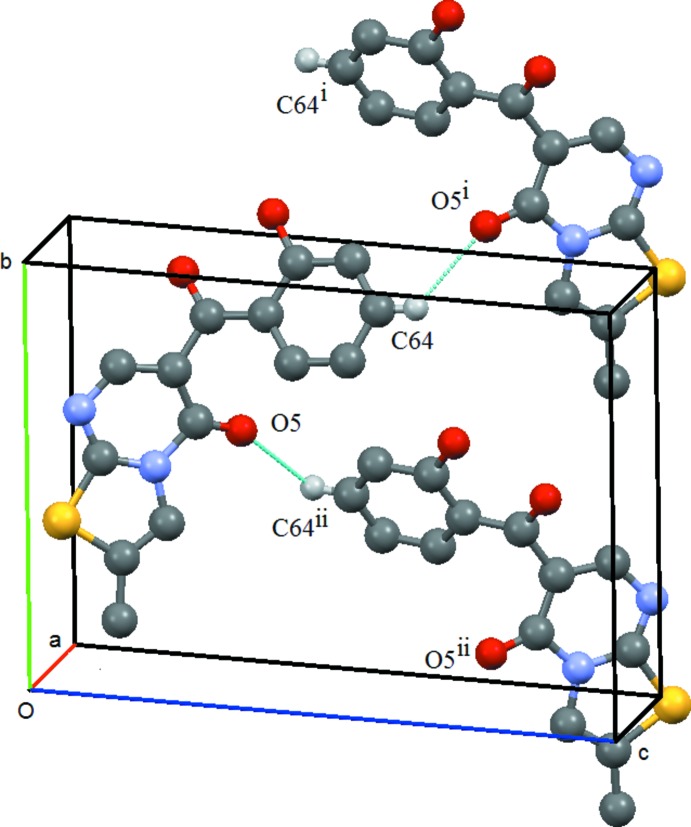
Compound (**2**): Mol­ecular *C*9 chain which runs parallel to the *a*-axis direction. Symmetry codes: (i) −*x* + 2, *y* + 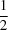, −*z* + 1; (ii) −*x* + 2, *y* − 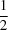, −*z* + 1. Hydrogen atoms not involved in the hydrogen bonding are omitted.

In (**1**) the mol­ecules are linked by the C2—H2⋯O5 (*x* + 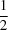, −*y* + 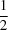, *z* + 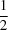) hydrogen bond, forming a *C*(6) chain, which runs parallel to [101] and results from the action of a *c*-glide at (0, 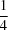, 0) (Table 1 ▸ and Fig. 3 ▸). The presence of the methyl group on atom C2 of the heterocyclic ring precludes the formation of a similar bond in (**2**). Thus in the supra­molecular structure of this compound, the mol­ecules are linked by a C64—H64⋯O5(−*x* + 2, *y* + 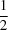, −*z* + 1) hydrogen bond, forming a *C*(9) chain, which runs parallel to the *b*-axis direction and results from the action of a 2_1_ screw axis at (1, *y*, 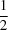) (Table 2 ▸ and Fig. 4 ▸).

Both mol­ecules present aromatic π–π stacking contacts. In (**1**) there is a close contact between centrosymmetrically related rings containing atom C5 at (*x*, *y*, *z*) and (−*x* + 1, −*y* + 1, −*z* + 1) [centroid-to-centroid distance = 3.6764 (9) Å, perpendicular distance between rings = 3.2478 (6) Å and slippage = 1.723 Å]. In (**2**) the mol­ecules stack above each other along the *a-*axis direction with unit translation of 3.931 (2) Å [perpendicular distances between the rings (and slippages) of 3.3821 (9) (2.004), 3.3355 (9) (2.080), 3.4084 (9) (1.958) Å for the thia­zole, pyrimidine and benzene rings, respectively].

## Database survey   

**Figure 5:**
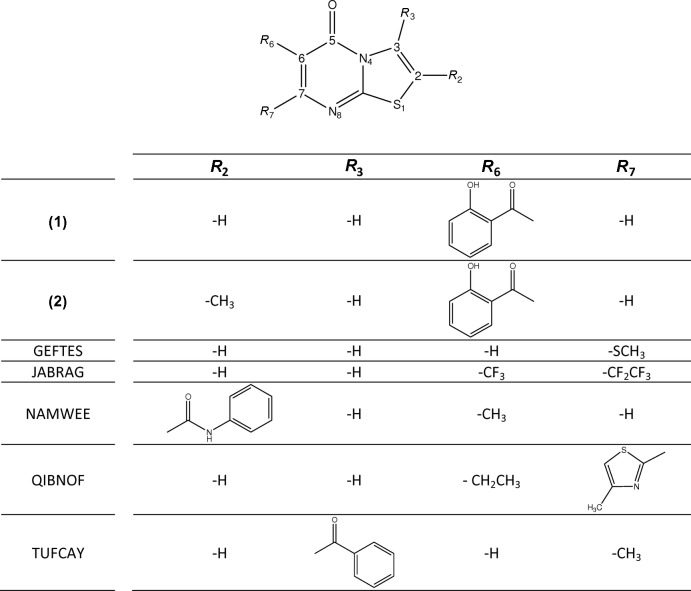
Representations of the compounds referred to in this work (the scaffold indicates the adopted numbering scheme for the 5*H*-thia­zolo[3,2*a*]-pyrimidine-5-one residue).

As said before, a search made in the latest version (5.36.0; 2015) of the Cambridge Structural Database revealed the existence of 11 deposited compounds containing the 5*H*-thia­zolo[3,2*a*]-pyrimidine-5-one residue. Of those, eight were 2,3-di­hydro derivatives thus leaving only the compounds listed below. Fig. 5 ▸ shows representations of the compounds referred to in this work (the scaffold indicates the adopted numbering scheme for the 5*H*-thia­zolo[3,2*a*]-pyrimidine-5-one residue). Compounds (**1**) and (**2**) are herein characterized and the remaining are referred to by their CSD codes. GEFTES: 7-(methyl­sulfan­yl)-5*H*-[1,3]thia­zolo[3,2-*a*]pyrimidin-5-one (Bernhardt & Wentrup, 2012 ▸); JABRAG: 7-penta­fluoro­ethyl-6-tri­fluoro­methyl­thia­zolo[3,2-*a*]pyrimidine-5-one (Chi *et al.*, 2002 ▸); NAMWEE: *N*-phenyl-6-methyl-5-oxo-5*H*-[1,3]-thia­zolo[3,2-*a*]pyrimidine-2-carboxamide (Volovenko *et al.*, 2004 ▸); QIBNOF: 3-ethyl-2-(4-methyl­thia­zol-2-yl)thia­zolo[3,2-*a*]pyrimidin-4-one (Troisi *et al.*, 2006 ▸); and TUFCAY: 3-benzoyl-7-methyl-5*H*-thia­zolo[3,2-*a*]pyrimidine-5-one (Elokhina *et al.*, 1996 ▸). In those compounds, the C2—C3 bond length averages 1.329 (9) Å, typical for values for a C*sp* ^2^—C*sp* ^2^ bond length in thio­phenes (Allen *et al.*, 1987 ▸). The average length of the C3—N4 bond at 1.397 (6) Å is slightly shorter than that for N4—C5, which is 1.418 (7) Å. The average values for the N4—C9 and C7—N8 bond lengths, 1.363 (7) and 1.357 (12) Å, respectively, are significantly shorter than the previous ones, suggesting the presence of a higher electronic density in that part of the rings. The N8—C9 average of 1.306 (9) Å is typical of a C=N bond.

## Synthesis and crystallization   

Compounds (**1**) and (**2**) were synthesized in moderate/high yields by a one-pot reaction using 4-oxo-4*H*-chromene-3-carb­oxy­lic acid as the starting material. Chromone-3-carb­oxy­lic acid was initially activated with benzotriazol-1-yl-oxy­tripyrrolidino­phospho­nium hexa­fluorido­phosphate (PyBOP). Then the *in situ* formed inter­mediate reacts with the hetero­amine (stoichiometry 1:1) giving rise to 5*H*-thia­zolo[3,2-*a*]pyrimidin-5-one derivatives (**1**) (68%) and (**2**) (81%). From a mechanistic point of view, the 6-(2-hy­droxy­benzo­yl)-5*H*-thia­zolo[3,2-*a*]pyrimidin-5-one derivatives may have been obtained by a nucleophilic attack of primary hetero­amine to the 2-position of the activated chromone with a subsequent opening of the pyran ring. Then, the heterocycle entities were obtained by a process involving an intra­molecular reaction assisted by the nitro­gen atom of the heterocycle moiety (see scheme below). Crystals were obtained by recrystallization from (**1**) in AcOEt (m.p. 454–456 K) in the form of colourless plates and from (**2**) in CH_2_Cl_2_ (m.p. 451–453 K) in the form of yellow blocks.
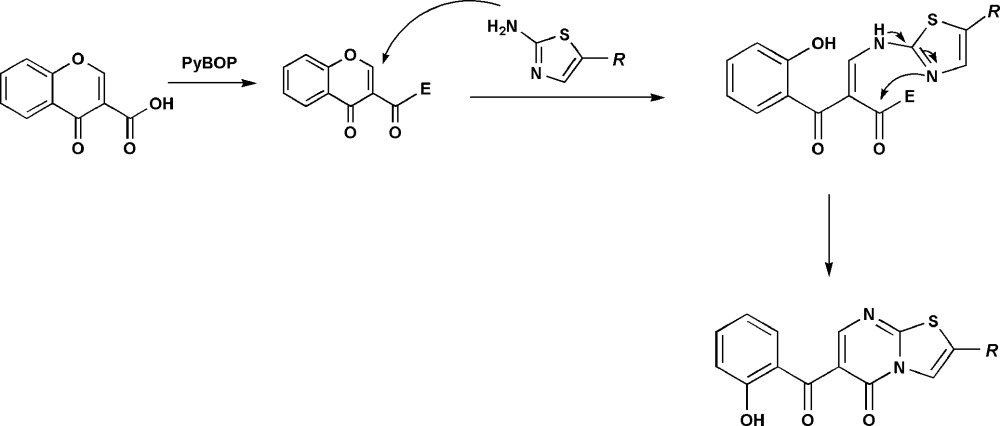


## Refinement   

Crystal data, data collection and structure refinement details are summarized in Table 3 ▸. H atoms were positioned geom­etrically and treated as riding atoms with C—H(aromatic) = 0.95 and O—H = 0.84 Å with *U*_iso_ = 1.2*U*_eq_(C) or 1.5*U*_eq_(O).

## Supplementary Material

Crystal structure: contains datablock(s) general, 1, 2. DOI: 10.1107/S2056989015011044/hb7437sup1.cif

Structure factors: contains datablock(s) 1. DOI: 10.1107/S2056989015011044/hb74371sup2.hkl

Structure factors: contains datablock(s) 2. DOI: 10.1107/S2056989015011044/hb74372sup3.hkl

Supporting information file. DOI: 10.1107/S2056989015011044/hb74371sup4.cml

Supporting information file. DOI: 10.1107/S2056989015011044/hb74372sup5.cml

CCDC references: 1405409, 1405408

Additional supporting information:  crystallographic information; 3D view; checkCIF report

## Figures and Tables

**Figure 2:**
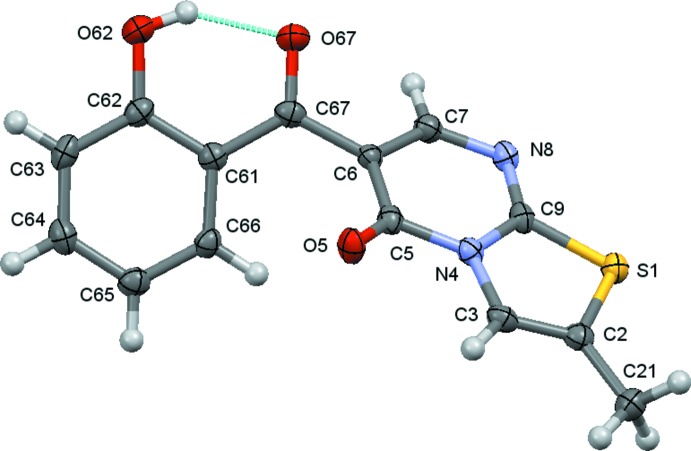
A view of the asymmetric unit of (**2**) with displacement ellipsoids drawn at the 70% probability level.

**Table 1:** Hydrogen-bond geometry (Å, °) for (**1**)

*D*—H⋯*A*	*D*—H	H⋯*A*	*D*⋯*A*	*D*—H⋯*A*
O62—H62*A*⋯O67	0.84	1.87	2.5906 (16)	144
C2—H2⋯O5^i^	0.95	2.29	3.146 (2)	150

Symmetry code: (i) 
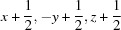
.

**Table 2:** Hydrogen-bond geometry (Å, °) for (**2**)

*D*—H⋯*A*	*D*—H	H⋯*A*	*D*⋯*A*	*D*—H⋯*A*
O62—H3⋯O67	0.84	1.81	2.557 (2)	146
C64—H64⋯O5^i^	0.95	2.57	3.217 (3)	125

Symmetry code: (i) 
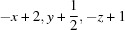
.

**Table 3:** Experimental details

	(**1**)	(**2**)
Crystal data		
Chemical formula	C_13_H_8_N_2_O_3_S	C_14_H_10_N_2_O_3_S
*M*_r_	272.27	286.30
Crystal system, space group	Monoclinic, *P*2_1_/*n*	Monoclinic, *P*2_1_
Temperature (K)	100	100
*a*, *b*, *c* (Å)	7.5563 (5), 15.3187 (11), 10.1229 (7)	3.931 (2), 10.459 (6), 14.657 (8)
β (°)	99.49 (2)	94.201 (14)
*V* (Å^3^)	1155.70 (15)	601.0 (6)
*Z*	4	2
Radiation type	Mo *K*α	Mo *K*α
μ (mm^−1^)	0.29	0.28
Crystal size (mm)	0.33 × 0.21 × 0.04	0.26 × 0.13 × 0.09

Data collection		
Diffractometer	Rigaku Saturn724+	Rigaku Saturn724+
Absorption correction	Multi-scan *CrystalClear-SM Expert* (Rigaku, 2012 ▸)	Multi-scan *CrystalClear-SM Expert* (Rigaku, 2012 ▸)
*T*_min_, *T*_max_	0.912, 0.989	0.931, 0.975
No. of measured, independent and observed [*I* > 2σ(*I*)] reflections	7713, 2632, 2135	4859, 3175, 2808
*R*_int_	0.040	0.023
(sin θ/λ)_max_ (Å^−1^)	0.649	0.729

Refinement		
*R*[*F* ^2^ > 2σ(*F* ^2^)], *wR*(*F* ^2^), *S*	0.036, 0.096, 1.02	0.031, 0.067, 1.04
No. of reflections	2632	3175
No. of parameters	172	183
No. of restraints	0	1
H-atom treatment	H-atom parameters constrained	H-atom parameters constrained
Δρ_max_, Δρ_min_ (e Å^−3^)	0.38, −0.22	0.35, −0.34
Absolute structure	–	Flack *x* determined using 981 quotients [(*I* ^+^)−(*I* ^−^)]/[(*I* ^+^)+(*I* ^−^)] (Parsons *et al.*, 2013 ▸)
Absolute structure parameter	–	−0.03 (4)

Computer programs: *CrystalClear-SM Expert* (Rigaku, 2012 ▸), *SHELXS* (Sheldrick, 2008 ▸), *ShelXle* (Hübschle *et al.*, 2011 ▸), *SHELXL2014* (Sheldrick, 2015 ▸), *PLATON* (Spek, 2009 ▸), *Flipper 25* (Oszlányi & Sütő, 2004 ▸), *OSCAIL* (McArdle *et al.*, 2004 ▸) and *Mercury* (Macrae *et al.*, 2006 ▸).

## supplementary crystallographic information

### (1) 6-(2-Hydroxybenzoyl)-5H-thiazolo[3,2-a]pyrimidin-5-one Crystal data

**Table:**

C_13_H_8_N_2_O_3_S	*F*(000) = 560
*M**_r_* = 272.27	*D*_x_ = 1.565 Mg m^−^^3^
Monoclinic, *P*2_1_/*n*	Mo *K*α radiation, λ = 0.71075 Å
*a* = 7.5563 (5) Å	Cell parameters from 7040 reflections
*b* = 15.3187 (11) Å	θ = 2.4–27.5°
*c* = 10.1229 (7) Å	µ = 0.28 mm^−^^1^
β = 99.49 (2)°	*T* = 100 K
*V* = 1155.70 (15) Å^3^	Plate, colourless
*Z* = 4	0.33 × 0.21 × 0.04 mm

### (1) 6-(2-Hydroxybenzoyl)-5H-thiazolo[3,2-a]pyrimidin-5-one Data collection

**Table:**

Rigaku Saturn724+ (2x2 bin mode) diffractometer	2632 independent reflections
Radiation source: Sealed Tube	2135 reflections with *I* > 2σ(*I*)
Graphite Monochromator monochromator	*R*_int_ = 0.040
Detector resolution: 28.5714 pixels mm^-1^	θ_max_ = 27.5°, θ_min_ = 3.1°
profile data from ω–scans	*h* = −8→9
Absorption correction: multi-scan *CrystalClear-SM Expert* (Rigaku, 2012)	*k* = −16→19
*T*_min_ = 0.912, *T*_max_ = 0.989	*l* = −13→10
7713 measured reflections	

### (1) 6-(2-Hydroxybenzoyl)-5H-thiazolo[3,2-a]pyrimidin-5-one Refinement

**Table:**

Refinement on *F*^2^	0 restraints
Least-squares matrix: full	Hydrogen site location: mixed
*R*[*F*^2^ > 2σ(*F*^2^)] = 0.036	H-atom parameters constrained
*wR*(*F*^2^) = 0.096	*w* = 1/[σ^2^(*F*_o_^2^) + (0.0485*P*)^2^ + 0.3293*P*] where *P* = (*F*_o_^2^ + 2*F*_c_^2^)/3
*S* = 1.02	(Δ/σ)_max_ = 0.001
2632 reflections	Δρ_max_ = 0.38 e Å^−^^3^
172 parameters	Δρ_min_ = −0.22 e Å^−^^3^

### (1) 6-(2-Hydroxybenzoyl)-5H-thiazolo[3,2-a]pyrimidin-5-one Special details

**Table:**

Geometry. All e.s.d.'s (except the e.s.d. in the dihedral angle between two l.s. planes) are estimated using the full covariance matrix. The cell e.s.d.'s are taken into account individually in the estimation of e.s.d.'s in distances, angles and torsion angles; correlations between e.s.d.'s in cell parameters are only used when they are defined by crystal symmetry. An approximate (isotropic) treatment of cell e.s.d.'s is used for estimating e.s.d.'s involving l.s. planes.

### (1) 6-(2-Hydroxybenzoyl)-5H-thiazolo[3,2-a]pyrimidin-5-one Fractional atomic coordinates and isotropic or equivalent isotropic displacement parameters (Å^2^)

**Table:**

	*x*	*y*	*z*	*U*_iso_*/*U*_eq_	
S1	0.53044 (5)	0.47242 (3)	0.82245 (4)	0.01896 (13)	
O5	0.13953 (16)	0.30924 (7)	0.48703 (11)	0.0220 (3)	
O62	−0.16684 (15)	0.43565 (8)	0.04276 (11)	0.0244 (3)	
H62A	−0.1549	0.4787	0.0949	0.037*	
O67	−0.03352 (15)	0.51893 (7)	0.25912 (11)	0.0202 (3)	
N4	0.32960 (17)	0.39593 (9)	0.62970 (12)	0.0153 (3)	
N8	0.35861 (18)	0.54953 (9)	0.59756 (13)	0.0182 (3)	
C2	0.4957 (2)	0.36098 (11)	0.83142 (15)	0.0200 (3)	
H2	0.5482	0.3254	0.9044	0.024*	
C3	0.3863 (2)	0.33073 (11)	0.72355 (15)	0.0184 (3)	
H3	0.3510	0.2713	0.7121	0.022*	
C5	0.2084 (2)	0.38137 (11)	0.50752 (15)	0.0167 (3)	
C6	0.1834 (2)	0.45847 (10)	0.42710 (14)	0.0157 (3)	
C7	0.2532 (2)	0.53721 (11)	0.47770 (15)	0.0174 (3)	
H7	0.2244	0.5873	0.4232	0.021*	
C9	0.3934 (2)	0.47688 (10)	0.66844 (15)	0.0159 (3)	
C61	0.1006 (2)	0.39010 (10)	0.19292 (14)	0.0162 (3)	
C62	−0.0209 (2)	0.38331 (11)	0.07150 (15)	0.0184 (3)	
C63	0.0072 (2)	0.32092 (11)	−0.02354 (16)	0.0219 (4)	
H62	−0.0774	0.3146	−0.1035	0.026*	
C64	0.1571 (2)	0.26854 (11)	−0.00138 (16)	0.0217 (4)	
H64	0.1752	0.2264	−0.0666	0.026*	
C65	0.2832 (2)	0.27646 (11)	0.11565 (16)	0.0201 (3)	
H65	0.3881	0.2413	0.1289	0.024*	
C66	0.2531 (2)	0.33623 (10)	0.21201 (15)	0.0178 (3)	
H66	0.3372	0.3409	0.2926	0.021*	
C67	0.0738 (2)	0.45816 (10)	0.29057 (15)	0.0160 (3)	

### (1) 6-(2-Hydroxybenzoyl)-5H-thiazolo[3,2-a]pyrimidin-5-one Atomic displacement parameters (Å^2^)

**Table:**

	*U*^11^	*U*^22^	*U*^33^	*U*^12^	*U*^13^	*U*^23^
S1	0.0213 (2)	0.0200 (2)	0.01334 (19)	−0.00121 (15)	−0.00382 (14)	0.00147 (15)
O5	0.0276 (6)	0.0188 (6)	0.0182 (6)	−0.0057 (5)	−0.0003 (5)	−0.0008 (4)
O62	0.0242 (6)	0.0272 (7)	0.0184 (6)	0.0057 (5)	−0.0068 (5)	−0.0042 (5)
O67	0.0210 (6)	0.0209 (6)	0.0167 (5)	0.0045 (5)	−0.0025 (4)	−0.0006 (4)
N4	0.0172 (6)	0.0143 (7)	0.0140 (6)	0.0011 (5)	0.0008 (5)	0.0005 (5)
N8	0.0209 (7)	0.0164 (7)	0.0151 (6)	0.0003 (5)	−0.0038 (5)	−0.0002 (5)
C2	0.0231 (8)	0.0204 (9)	0.0160 (7)	0.0034 (6)	0.0019 (6)	0.0034 (6)
C3	0.0235 (8)	0.0150 (8)	0.0166 (7)	0.0028 (6)	0.0033 (6)	0.0039 (6)
C5	0.0161 (7)	0.0188 (9)	0.0148 (7)	−0.0002 (6)	0.0015 (6)	−0.0025 (6)
C6	0.0164 (7)	0.0177 (9)	0.0122 (7)	0.0015 (6)	−0.0004 (6)	−0.0016 (6)
C7	0.0181 (7)	0.0174 (9)	0.0154 (7)	0.0019 (6)	−0.0014 (6)	0.0008 (6)
C9	0.0158 (7)	0.0156 (8)	0.0153 (7)	0.0001 (6)	−0.0007 (6)	−0.0009 (6)
C61	0.0179 (7)	0.0161 (8)	0.0136 (7)	−0.0025 (6)	0.0002 (6)	−0.0002 (6)
C62	0.0193 (7)	0.0181 (9)	0.0164 (7)	−0.0009 (6)	−0.0013 (6)	0.0013 (6)
C63	0.0283 (8)	0.0211 (9)	0.0145 (7)	−0.0042 (7)	−0.0017 (6)	−0.0004 (6)
C64	0.0328 (9)	0.0163 (9)	0.0165 (7)	−0.0029 (7)	0.0059 (7)	−0.0021 (6)
C65	0.0241 (8)	0.0163 (9)	0.0200 (8)	0.0015 (6)	0.0043 (6)	0.0006 (6)
C66	0.0196 (8)	0.0171 (9)	0.0160 (7)	−0.0015 (6)	0.0007 (6)	0.0005 (6)
C67	0.0153 (7)	0.0162 (8)	0.0158 (7)	−0.0014 (6)	0.0004 (6)	0.0016 (6)

### (1) 6-(2-Hydroxybenzoyl)-5H-thiazolo[3,2-a]pyrimidin-5-one Geometric parameters (Å, º)

**Table:**

S1—C9	1.7248 (16)	C6—C7	1.381 (2)
S1—C2	1.7318 (17)	C6—C67	1.489 (2)
O5—C5	1.225 (2)	C7—H7	0.9500
O62—C62	1.3559 (19)	C61—C66	1.404 (2)
O62—H62A	0.8405	C61—C62	1.411 (2)
O67—C67	1.2413 (19)	C61—C67	1.473 (2)
N4—C9	1.364 (2)	C62—C63	1.397 (2)
N4—C3	1.396 (2)	C63—C64	1.376 (2)
N4—C5	1.4298 (19)	C63—H62	0.9500
N8—C9	1.327 (2)	C64—C65	1.397 (2)
N8—C7	1.3503 (19)	C64—H64	0.9500
C2—C3	1.339 (2)	C65—C66	1.384 (2)
C2—H2	0.9500	C65—H65	0.9500
C3—H3	0.9500	C66—H66	0.9500
C5—C6	1.429 (2)		
			
C9—S1—C2	90.74 (7)	N4—C9—S1	110.78 (11)
C62—O62—H62A	109.3	C66—C61—C62	118.53 (14)
C9—N4—C3	113.61 (13)	C66—C61—C67	121.59 (14)
C9—N4—C5	122.40 (13)	C62—C61—C67	119.65 (14)
C3—N4—C5	123.91 (13)	O62—C62—C63	117.85 (14)
C9—N8—C7	113.80 (14)	O62—C62—C61	122.18 (14)
C3—C2—S1	112.14 (12)	C63—C62—C61	119.97 (15)
C3—C2—H2	123.9	C64—C63—C62	120.06 (15)
S1—C2—H2	123.9	C64—C63—H62	120.0
C2—C3—N4	112.72 (15)	C62—C63—H62	120.0
C2—C3—H3	123.6	C63—C64—C65	121.00 (15)
N4—C3—H3	123.6	C63—C64—H64	119.5
O5—C5—N4	118.75 (14)	C65—C64—H64	119.5
O5—C5—C6	129.54 (14)	C66—C65—C64	119.14 (15)
N4—C5—C6	111.69 (13)	C66—C65—H65	120.4
C7—C6—C5	120.26 (14)	C64—C65—H65	120.4
C7—C6—C67	117.84 (14)	C65—C66—C61	121.21 (15)
C5—C6—C67	121.81 (13)	C65—C66—H66	119.4
N8—C7—C6	126.02 (15)	C61—C66—H66	119.4
N8—C7—H7	117.0	O67—C67—C61	120.99 (14)
C6—C7—H7	117.0	O67—C67—C6	118.38 (14)
N8—C9—N4	125.30 (14)	C61—C67—C6	120.53 (13)
N8—C9—S1	123.91 (12)		
			
C9—S1—C2—C3	−0.18 (13)	C2—S1—C9—N8	−179.59 (14)
S1—C2—C3—N4	0.97 (18)	C2—S1—C9—N4	−0.67 (12)
C9—N4—C3—C2	−1.52 (19)	C66—C61—C62—O62	−176.96 (15)
C5—N4—C3—C2	−178.23 (13)	C67—C61—C62—O62	−2.4 (2)
C9—N4—C5—O5	−171.08 (14)	C66—C61—C62—C63	3.3 (2)
C3—N4—C5—O5	5.4 (2)	C67—C61—C62—C63	177.80 (14)
C9—N4—C5—C6	7.34 (19)	O62—C62—C63—C64	177.45 (15)
C3—N4—C5—C6	−176.23 (13)	C61—C62—C63—C64	−2.8 (2)
O5—C5—C6—C7	170.03 (16)	C62—C63—C64—C65	0.1 (3)
N4—C5—C6—C7	−8.2 (2)	C63—C64—C65—C66	1.9 (2)
O5—C5—C6—C67	−6.4 (3)	C64—C65—C66—C61	−1.4 (2)
N4—C5—C6—C67	175.39 (13)	C62—C61—C66—C65	−1.2 (2)
C9—N8—C7—C6	0.0 (2)	C67—C61—C66—C65	−175.63 (14)
C5—C6—C7—N8	5.1 (2)	C66—C61—C67—O67	161.58 (15)
C67—C6—C7—N8	−178.29 (14)	C62—C61—C67—O67	−12.8 (2)
C7—N8—C9—N4	−1.1 (2)	C66—C61—C67—C6	−14.7 (2)
C7—N8—C9—S1	177.67 (11)	C62—C61—C67—C6	170.93 (14)
C3—N4—C9—N8	−179.75 (14)	C7—C6—C67—O67	−40.9 (2)
C5—N4—C9—N8	−3.0 (2)	C5—C6—C67—O67	135.67 (15)
C3—N4—C9—S1	1.35 (16)	C7—C6—C67—C61	135.52 (15)
C5—N4—C9—S1	178.12 (10)	C5—C6—C67—C61	−48.0 (2)

### (1) 6-(2-Hydroxybenzoyl)-5H-thiazolo[3,2-a]pyrimidin-5-one Hydrogen-bond geometry (Å, º)

**Table:**

*D*—H···*A*	*D*—H	H···*A*	*D*···*A*	*D*—H···*A*
O62—H62*A*···O67	0.84	1.87	2.5906 (16)	144
C2—H2···O5^i^	0.95	2.29	3.146 (2)	150

Symmetry code: (i) *x*+1/2, −*y*+1/2, *z*+1/2.

### (2) 6-(2-Hydroxybenzoyl)-2-methyl-5H-thiazolo[3,2-a]pyrimidin-5-one Crystal data

**Table:**

C_14_H_10_N_2_O_3_S	*F*(000) = 296
*M**_r_* = 286.30	*D*_x_ = 1.582 Mg m^−^^3^
Monoclinic, *P*2_1_	Mo *K*α radiation, λ = 0.71075 Å
*a* = 3.931 (2) Å	Cell parameters from 1337 reflections
*b* = 10.459 (6) Å	θ = 2.4–31.1°
*c* = 14.657 (8) Å	µ = 0.28 mm^−^^1^
β = 94.201 (14)°	*T* = 100 K
*V* = 601.0 (6) Å^3^	Block, yellow
*Z* = 2	0.26 × 0.13 × 0.09 mm

### (2) 6-(2-Hydroxybenzoyl)-2-methyl-5H-thiazolo[3,2-a]pyrimidin-5-one Data collection

**Table:**

Rigaku Saturn724+ (2x2 bin mode) diffractometer	3175 independent reflections
Radiation source: Rotating Anode	2808 reflections with *I* > 2σ(*I*)
Confocal monochromator	*R*_int_ = 0.023
Detector resolution: 28.5714 pixels mm^-1^	θ_max_ = 31.2°, θ_min_ = 2.4°
profile data from ω–scans	*h* = −5→5
Absorption correction: multi-scan *CrystalClear-SM Expert* (Rigaku, 2012)	*k* = −14→14
*T*_min_ = 0.931, *T*_max_ = 0.975	*l* = −18→20
4859 measured reflections	

### (2) 6-(2-Hydroxybenzoyl)-2-methyl-5H-thiazolo[3,2-a]pyrimidin-5-one Refinement

**Table:**

Refinement on *F*^2^	Hydrogen site location: inferred from neighbouring sites
Least-squares matrix: full	H-atom parameters constrained
*R*[*F*^2^ > 2σ(*F*^2^)] = 0.031	*w* = 1/[σ^2^(*F*_o_^2^) + (0.0296*P*)^2^] where *P* = (*F*_o_^2^ + 2*F*_c_^2^)/3
*wR*(*F*^2^) = 0.067	(Δ/σ)_max_ < 0.001
*S* = 1.04	Δρ_max_ = 0.35 e Å^−^^3^
3175 reflections	Δρ_min_ = −0.33 e Å^−^^3^
183 parameters	Absolute structure: Flack *x* determined using 981 quotients [(*I*^+^)-(*I*^-^)]/[(*I*^+^)+(*I*^-^)] (Parsons *et al.*, 2013)
1 restraint	Absolute structure parameter: −0.03 (4)

### (2) 6-(2-Hydroxybenzoyl)-2-methyl-5H-thiazolo[3,2-a]pyrimidin-5-one Special details

**Table:**

Geometry. All e.s.d.'s (except the e.s.d. in the dihedral angle between two l.s. planes) are estimated using the full covariance matrix. The cell e.s.d.'s are taken into account individually in the estimation of e.s.d.'s in distances, angles and torsion angles; correlations between e.s.d.'s in cell parameters are only used when they are defined by crystal symmetry. An approximate (isotropic) treatment of cell e.s.d.'s is used for estimating e.s.d.'s involving l.s. planes.

### (2) 6-(2-Hydroxybenzoyl)-2-methyl-5H-thiazolo[3,2-a]pyrimidin-5-one Fractional atomic coordinates and isotropic or equivalent isotropic displacement parameters (Å^2^)

**Table:**

	*x*	*y*	*z*	*U*_iso_*/*U*_eq_	
S1	0.15253 (13)	0.38511 (5)	0.04641 (4)	0.01618 (13)	
O62	0.9984 (4)	1.04996 (14)	0.34842 (12)	0.0214 (4)	
H3	0.9814	1.0301	0.2927	0.032*	
O5	0.8745 (4)	0.55020 (15)	0.29853 (11)	0.0183 (3)	
O67	0.8149 (4)	0.91654 (14)	0.20714 (11)	0.0197 (4)	
N4	0.4972 (4)	0.49139 (17)	0.17956 (13)	0.0138 (4)	
N8	0.2366 (5)	0.63883 (18)	0.07249 (13)	0.0159 (4)	
C2	0.3438 (5)	0.2885 (2)	0.13351 (16)	0.0155 (4)	
C3	0.5170 (5)	0.3595 (2)	0.19717 (15)	0.0155 (5)	
H3A	0.6412	0.3241	0.2492	0.019*	
C5	0.6695 (5)	0.5856 (2)	0.23699 (16)	0.0146 (4)	
C6	0.5742 (5)	0.7132 (2)	0.20851 (15)	0.0129 (4)	
C7	0.3746 (5)	0.7315 (2)	0.12859 (16)	0.0156 (4)	
H7	0.3288	0.8175	0.1108	0.019*	
C9	0.3047 (5)	0.5225 (2)	0.10207 (15)	0.0143 (4)	
C21	0.3011 (6)	0.1472 (2)	0.13112 (17)	0.0196 (5)	
H21A	0.4177	0.1097	0.1861	0.029*	
H21B	0.3997	0.1128	0.0767	0.029*	
H21C	0.0578	0.1260	0.1289	0.029*	
C61	0.7116 (5)	0.8455 (2)	0.35577 (15)	0.0142 (4)	
C62	0.8621 (5)	0.9562 (2)	0.39773 (16)	0.0157 (5)	
C63	0.8759 (5)	0.9700 (2)	0.49229 (17)	0.0177 (5)	
H63	0.9829	1.0429	0.5205	0.021*	
C64	0.7340 (5)	0.8779 (2)	0.54512 (15)	0.0182 (4)	
H64	0.7464	0.8878	0.6097	0.022*	
C65	0.5725 (5)	0.7705 (2)	0.50554 (16)	0.0174 (5)	
H65	0.4721	0.7087	0.5427	0.021*	
C66	0.5602 (5)	0.7550 (2)	0.41204 (15)	0.0151 (4)	
H66	0.4483	0.6824	0.3849	0.018*	
C67	0.7112 (5)	0.8297 (2)	0.25681 (15)	0.0150 (4)	

### (2) 6-(2-Hydroxybenzoyl)-2-methyl-5H-thiazolo[3,2-a]pyrimidin-5-one Atomic displacement parameters (Å^2^)

**Table:**

	*U*^11^	*U*^22^	*U*^33^	*U*^12^	*U*^13^	*U*^23^
S1	0.0175 (2)	0.0166 (3)	0.0143 (2)	−0.0011 (2)	−0.00033 (18)	−0.0012 (2)
O62	0.0274 (8)	0.0165 (8)	0.0203 (9)	−0.0060 (7)	0.0026 (7)	0.0000 (7)
O5	0.0213 (8)	0.0179 (8)	0.0148 (8)	0.0035 (7)	−0.0049 (7)	−0.0009 (7)
O67	0.0249 (8)	0.0161 (9)	0.0182 (8)	−0.0015 (6)	0.0026 (6)	0.0025 (6)
N4	0.0149 (8)	0.0143 (10)	0.0121 (9)	0.0016 (7)	0.0009 (7)	0.0007 (7)
N8	0.0173 (9)	0.0180 (10)	0.0124 (9)	0.0006 (8)	−0.0001 (7)	−0.0003 (8)
C2	0.0154 (10)	0.0164 (11)	0.0150 (11)	−0.0002 (9)	0.0032 (8)	0.0009 (9)
C3	0.0165 (9)	0.0159 (12)	0.0144 (11)	0.0029 (8)	0.0025 (8)	0.0032 (8)
C5	0.0138 (9)	0.0173 (11)	0.0131 (11)	−0.0003 (9)	0.0026 (8)	−0.0023 (9)
C6	0.0157 (9)	0.0119 (10)	0.0114 (11)	0.0004 (8)	0.0033 (8)	0.0001 (8)
C7	0.0178 (10)	0.0147 (11)	0.0144 (11)	0.0016 (9)	0.0027 (8)	0.0010 (8)
C9	0.0124 (9)	0.0179 (11)	0.0123 (11)	0.0008 (9)	0.0000 (8)	−0.0023 (9)
C21	0.0220 (11)	0.0163 (11)	0.0204 (13)	−0.0019 (10)	0.0011 (9)	−0.0017 (10)
C61	0.0138 (9)	0.0138 (11)	0.0150 (11)	0.0020 (8)	0.0008 (8)	0.0002 (8)
C62	0.0144 (9)	0.0134 (11)	0.0193 (12)	0.0009 (8)	0.0009 (8)	−0.0001 (8)
C63	0.0168 (10)	0.0145 (11)	0.0215 (12)	0.0003 (9)	−0.0018 (9)	−0.0048 (9)
C64	0.0189 (9)	0.0211 (11)	0.0144 (10)	0.0038 (12)	−0.0006 (8)	−0.0022 (11)
C65	0.0188 (10)	0.0142 (11)	0.0191 (11)	0.0017 (9)	0.0015 (9)	0.0016 (9)
C66	0.0168 (10)	0.0117 (10)	0.0166 (11)	0.0012 (8)	0.0006 (8)	−0.0012 (8)
C67	0.0148 (10)	0.0142 (10)	0.0160 (11)	0.0029 (8)	0.0011 (9)	0.0030 (9)

### (2) 6-(2-Hydroxybenzoyl)-2-methyl-5H-thiazolo[3,2-a]pyrimidin-5-one Geometric parameters (Å, º)

**Table:**

S1—C9	1.737 (2)	C6—C67	1.490 (3)
S1—C2	1.754 (2)	C7—H7	0.9500
O62—C62	1.352 (3)	C21—H21A	0.9800
O62—H3	0.8400	C21—H21B	0.9800
O5—C5	1.222 (3)	C21—H21C	0.9800
O67—C67	1.251 (3)	C61—C66	1.415 (3)
N4—C9	1.357 (3)	C61—C62	1.419 (3)
N4—C3	1.404 (3)	C61—C67	1.460 (3)
N4—C5	1.434 (3)	C62—C63	1.391 (3)
N8—C9	1.313 (3)	C63—C64	1.379 (3)
N8—C7	1.358 (3)	C63—H63	0.9500
C2—C3	1.339 (3)	C64—C65	1.396 (3)
C2—C21	1.487 (3)	C64—H64	0.9500
C3—H3A	0.9500	C65—C66	1.377 (3)
C5—C6	1.439 (3)	C65—H65	0.9500
C6—C7	1.375 (3)	C66—H66	0.9500
			
C9—S1—C2	91.17 (12)	H21A—C21—H21B	109.5
C62—O62—H3	109.5	C2—C21—H21C	109.5
C9—N4—C3	114.16 (19)	H21A—C21—H21C	109.5
C9—N4—C5	122.48 (18)	H21B—C21—H21C	109.5
C3—N4—C5	123.4 (2)	C66—C61—C62	118.2 (2)
C9—N8—C7	113.5 (2)	C66—C61—C67	122.2 (2)
C3—C2—C21	128.2 (2)	C62—C61—C67	119.57 (19)
C3—C2—S1	110.87 (17)	O62—C62—C63	118.0 (2)
C21—C2—S1	120.92 (18)	O62—C62—C61	121.9 (2)
C2—C3—N4	113.5 (2)	C63—C62—C61	120.10 (19)
C2—C3—H3A	123.3	C64—C63—C62	119.9 (2)
N4—C3—H3A	123.3	C64—C63—H63	120.0
O5—C5—N4	118.9 (2)	C62—C63—H63	120.0
O5—C5—C6	129.7 (2)	C63—C64—C65	121.3 (2)
N4—C5—C6	111.41 (19)	C63—C64—H64	119.4
C7—C6—C5	119.8 (2)	C65—C64—H64	119.4
C7—C6—C67	117.0 (2)	C66—C65—C64	119.3 (2)
C5—C6—C67	122.88 (19)	C66—C65—H65	120.3
N8—C7—C6	126.4 (2)	C64—C65—H65	120.3
N8—C7—H7	116.8	C65—C66—C61	121.1 (2)
C6—C7—H7	116.8	C65—C66—H66	119.5
N8—C9—N4	125.9 (2)	C61—C66—H66	119.5
N8—C9—S1	123.84 (17)	O67—C67—C61	121.3 (2)
N4—C9—S1	110.26 (16)	O67—C67—C6	116.06 (19)
C2—C21—H21A	109.5	C61—C67—C6	122.63 (18)
C2—C21—H21B	109.5		
			
C9—S1—C2—C3	−1.90 (17)	C5—N4—C9—S1	176.89 (15)
C9—S1—C2—C21	177.56 (18)	C2—S1—C9—N8	−177.19 (19)
C21—C2—C3—N4	−178.26 (19)	C2—S1—C9—N4	2.17 (15)
S1—C2—C3—N4	1.1 (2)	C66—C61—C62—O62	−176.97 (19)
C9—N4—C3—C2	0.5 (3)	C67—C61—C62—O62	1.8 (3)
C5—N4—C3—C2	−178.30 (19)	C66—C61—C62—C63	3.7 (3)
C9—N4—C5—O5	−170.18 (19)	C67—C61—C62—C63	−177.5 (2)
C3—N4—C5—O5	8.6 (3)	O62—C62—C63—C64	178.71 (19)
C9—N4—C5—C6	7.7 (3)	C61—C62—C63—C64	−1.9 (3)
C3—N4—C5—C6	−173.54 (18)	C62—C63—C64—C65	−0.6 (3)
O5—C5—C6—C7	170.2 (2)	C63—C64—C65—C66	1.2 (3)
N4—C5—C6—C7	−7.4 (3)	C64—C65—C66—C61	0.7 (3)
O5—C5—C6—C67	−3.2 (4)	C62—C61—C66—C65	−3.1 (3)
N4—C5—C6—C67	179.16 (18)	C67—C61—C66—C65	178.16 (19)
C9—N8—C7—C6	1.3 (3)	C66—C61—C67—O67	172.6 (2)
C5—C6—C7—N8	3.4 (3)	C62—C61—C67—O67	−6.1 (3)
C67—C6—C7—N8	177.25 (19)	C66—C61—C67—C6	−4.5 (3)
C7—N8—C9—N4	−1.1 (3)	C62—C61—C67—C6	176.78 (18)
C7—N8—C9—S1	178.13 (16)	C7—C6—C67—O67	−40.7 (3)
C3—N4—C9—N8	177.37 (19)	C5—C6—C67—O67	133.0 (2)
C5—N4—C9—N8	−3.8 (3)	C7—C6—C67—C61	136.6 (2)
C3—N4—C9—S1	−2.0 (2)	C5—C6—C67—C61	−49.8 (3)

### (2) 6-(2-Hydroxybenzoyl)-2-methyl-5H-thiazolo[3,2-a]pyrimidin-5-one Hydrogen-bond geometry (Å, º)

**Table:**

*D*—H···*A*	*D*—H	H···*A*	*D*···*A*	*D*—H···*A*
O62—H3···O67	0.84	1.81	2.557 (2)	146
C64—H64···O5^i^	0.95	2.57	3.217 (3)	125

Symmetry code: (i) −*x*+2, *y*+1/2, −*z*+1.
